# Fabrication and Characterization of Double- and Single-Clamped CuO Nanowire Based Nanoelectromechanical Switches

**DOI:** 10.3390/nano11010117

**Published:** 2021-01-06

**Authors:** Liga Jasulaneca, Alexander I. Livshits, Raimonds Meija, Jelena Kosmaca, Raitis Sondors, Matiss M. Ramma, Daniels Jevdokimovs, Juris Prikulis, Donats Erts

**Affiliations:** 1 Institute of Chemical Physics, University of Latvia, 1 Jelgavas Street, LV-1004 Riga, Latvia; liga.jasulaneca@lu.lv (L.J.); aleksandrs.livsics@lu.lv (A.I.L.); raimonds.meija@lu.lv (R.M.); jelena.kosmaca@lu.lv (J.K.); raitis.sondors@lu.lv (R.S.); matiss_martins.ramma@lu.lv (M.M.R.); daniels.jevdokimovs@lu.lv (D.J.); juris.prikulis@lu.lv (J.P.); 2 Faculty of Chemistry, University of Latvia, 1 Jelgavas Street, LV-1004 Riga, Latvia

**Keywords:** nanoelectromechanical switch, NEMS, nanowires, bottom-up, CuO

## Abstract

Electrostatically actuated nanoelectromechanical (NEM) switches hold promise for operation with sharply defined ON/OFF states, high ON/OFF current ratio, low OFF state power consumption, and a compact design. The present challenge for the development of nanoelectromechanical system (NEMS) technology is fabrication of single nanowire based NEM switches. In this work, we demonstrate the first application of CuO nanowires as NEM switch active elements. We develop bottom-up and top-down approaches for NEM switch fabrication, such as CuO nanowire synthesis, lithography, etching, dielectrophoretic alignment of nanowires on electrodes, and nanomanipulations for building devices that are suitable for scalable production. Theoretical modelling finds the device geometry that is necessary for volatile switching. The modelling results are validated by constructing gateless double-clamped and single-clamped devices on-chip that show robust and repeatable switching. The proposed design and fabrication route enable the scalable integration of bottom-up synthesized nanowires in NEMS.

## 1. Introduction

The nanoelectromechanical (NEM) switch stands out as an energy-efficient candidate for logic and memory applications, due to two most important characteristics of mechanical switching: well-defined ON and OFF states and zero OFF state current [1,2,3], thus making it the ideal switch. Because atomic diffusion does not significantly impact the performance of these devices, mechanical switches can withstand a higher temperature than their conventional electronic-only semiconductor counterparts [2,4]. Despite the promising characteristics, the scalable fabrication and reliability of NEM switches is still an ongoing effort from both the characterization and technological perspectives [1,2].

The operation and properties of NEM switches can be experimentally explored by two approaches: using nanomanipulations in situ inside transmission [5,6,7,8,9] or scanning [10,11,12] electron microscopes and as micro-/nanofabricated on-chip devices [13,14,15,16,17,18].

The ability of rapid adjustment of the NEM switch configuration is an advantage of the in situ electron microscopy approach while using nanomanipulations [10,11,12], which allows for exploring different working regimes without the repetitive nanofabrication of multiple devices. Novel methods for NEM switch operation have been previously demonstrated in situ, such as decreasing of the switch-ON and switch-OFF voltages by using oscillations at resonance frequencies [10,12]. In situ characterization inside TEM and SEM have also allowed for the determination of the mechanical properties of NEM switch active elements, such as Young’s modulus [5,19,20,21], breaking strength [5], and resonant behavior [21,22].

The first on-chip devices were implemented while using carbon nanotube (CNT)-based nanorelays [13], followed by different metals [14,23], semiconductors [17,18], and, recently, also TiN ceramics [15] and Cu [16]. Most efforts in the fabrication of NEM switches have focused on the top-down approach, integrating the fabrication of the active element in a complex process flow [4,14,15,24]. However, this constrains the available materials and design architectures that are compatible with lithographic processing. The alternative use of bottom-up synthesized quasi-one-dimensional (1D) nanostructures (nanotubes, nanowires) [10,12,25] taps into an exciting field of various materials with different properties and versatile functionality for application in NEM switches.

Among the bottom-up synthesized 1D structures, CNTs are, by far, the most popular choice for NEM switches, due to their high Young’s modulus [25] and high resonance frequencies [26]. However, CNT-based NEM switches suffer from ablation during contact and they require additional electrode coating (e.g., diamond-like-carbon) to enhance contact reliability [27]. Single-crystalline and defect-free semiconductor nanowires [28], such as Si [17], Ge [8,29], and GeSn [12], have also been explored as potential candidate materials and demonstrated reliable operation at voltages up to 40–50 V. This enables their applications for high-voltage and low-current devices. CuO is a narrow band gap semiconductor that can be thermally oxidized in order to produce nanowires. It has been recently shown that CuO nanowires exhibit mechanical and electrical properties that are suitable for NEM switch applications [30,31]. High yield, controllable morphology of the as-grown nanowires and the low cost and simplicity of the method motivate the investigation of CuO nanowire use in NEM switches.

One of the greatest challenges for NEM switch fabrication while using bottom-up methods is the precise positioning of the active elements relative to the metal electrodes. The assembly of bottom-up synthesized active elements for NEM switches can be realized by spin-coating of the nanowire suspension [32], using dry mechanical transfer [33], dielectrophoresis (DEP) [34,35,36], and mechanical manipulation [37,38]. Dry mechanical transfer by pressing the substrate with nanowires against the chip with electrodes is fast and cost-effective, but it cannot ensure the nanowire positioning in predefined locations. Mechanical manipulation allows for precise alignment, but it is very time consuming. The DEP technique that is used in the present study increases the device fabrication throughput significantly by careful tuning of process parameters. The suspended length of the nanowire, number of nanowires, and their alignment direction on the electrodes can be efficiently controlled by DEP parameters (frequency, voltage, time), hydrodynamic forces, distance between the electrodes, and their shape and size [34,35].

To the best of our knowledge, integration of bottom-up synthesized semiconductor nanowires in NEM switches on-chip and their operation has not yet been tested. Here, we develop a theoretical model for optimizing the device parameters for volatile NEM switch operation. The model is used to calculate the switch-ON voltages for different NEM switch dimensions. Based on the acquired theoretical results, the full fabrication process of single- and double-clamped NEM switch is performed: (1) a scalable cost effective synthesis of CuO nanowires [30] for use as the NEM switch active elements; (2) fabrication of micropatterned device electrodes; and (3) dielectrophoresis [33] to align the nanowires on the electrodes. The developed NEM switches are tested for volatile switching in order to validate the theoretical results for both double-clamped and single-clamped devices.

## 2. Materials and Methods

**Figure 1:**
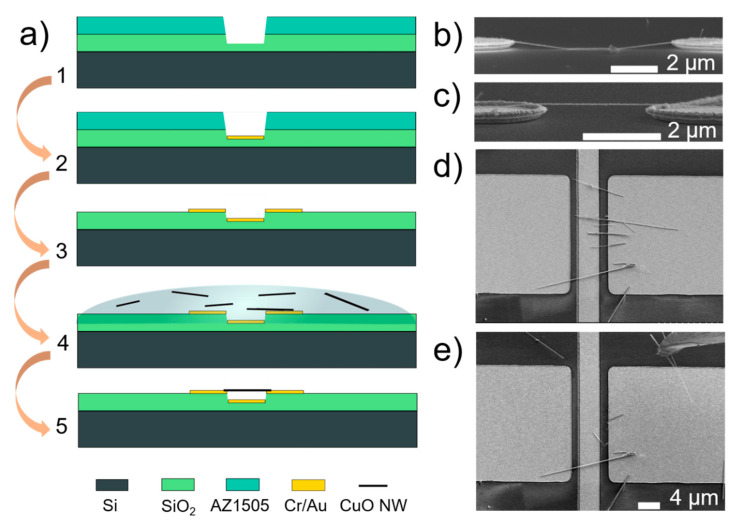
(**a**) Process flow for fabrication of microelectrodes involving etching of thermal SiO_2_ (1), deposition of metal electrodes (2, 3) and dielectrophoretic assembly and supercritical drying (4) to yield the final suspended device (5). SEM images showing side view of a single nanowire aligned on 2 µm wide electrodes by (**b**) DEP without supercritical drying, (**c**) dielectrophoresis (DEP) followed by supercritical drying; (**d**) SEM image showing top-view of multiple nanowires aligned on 20 µm wide electrodes; and, (**e**) the removal of excess nanowires by nanomanipulations with etched gold tip (tip shown in upper right side of the image).

Copper oxide (CuO) nanowires were synthesized by thermal oxidation of a Cu foil (99.9% purity, 25 μm thickness; Goodfellow GmbH, Hamburg, Germany) in a tube furnace (GSL-1100X, MTI Corporation, Richmond, CA, USA) at 500 °C for 3.5 h in ambient air, as reported in [30,39]. Figure 1a shows the typical process flow for the fabrication of electrodes and alignment of the as-synthesized nanowires. For microelectrode fabrication, AZ 1505 photoresist (Microchemicals GmbH, Ulm, Germany) was spin coated on a silicon substrate with thermal silicon dioxide (Microchemicals GmbH, Ulm, Germany) and then patterned using maskless optical lithography direct write system (Heidelberg µPG 101 Micro Pattern Generator, Heidelberg Instruments Mikrotechnik GmbH, Heidelberg, Germany) with a 375 nm laser source by the following procedure. Si substrates with 1000 nm thick thermal SiO_2_ were cleaned, and then a pattern was defined in a first photoresist layer. After post-bake of the photoresist, the exposed SiO_2_ was etched with a commercially available buffered oxide etch (BOE 7:1 with Surfactant; Microchemicals GmbH, Ulm, Germany) (Figure 1(a1)) in order to define the distance between the active element and the electrode. 10 nm Cr adhesion layer/70 nm Au layer (Kurt J. Lesker Company, Jefferson Hills, PA, USA) was thermally evaporated into the etched trenches (Figure 1(a2)) to obtain actuation electrodes. After lift-off, the second layer was aligned and patterned while using metal evaporation and lift-off (Figure 1(a3)) in order to define DEP electrodes. Nanowires were assembled on microelectrodes while using dielectrophoretic alignment (Figure 1(a4)) to yield final devices with nanowires that were suspended over a gold electrode (Figure 1(a5)).

To remove the nanowires from the oxidized Cu foil, the sample was placed in isopropyl alcohol and then ultrasonicated for 3 s. For DEP alignment, the as-fabricated chips were immersed in a nanowire-isopropanol suspension, applying AC signal with frequency of 50 kHz [33]. A floating electrode DEP configuration [40,41] was employed, which consisted of an array of two opposing electrodes. One electrode was connected to a common line to which the AC signal was applied. The opposite DEP electrode was held at a floating potential. AC ground was connected to the back of the silicon substrate. Separate actuation electrodes were fabricated between pairs of DEP electrodes, so that the voltage bias could be individually applied to each single-nanowire device. This design allowed for decoupling and analyzing single device characteristics from the DEP array without parallel interference from the other devices.

For the alignment of single nanowires in a double-clamped configuration, DEP was performed on 2 µm wide tapered electrodes (Figure 1b,c). In order to increase the yield of suspended nanowires, 20 µm wide electrodes for multiple nanowire DEP alignment in both single-clamped and double-clamped configurations were used (Figure 1d,e). Finally, to avoid nanowire’s stiction by capillary forces to the substrate during drying (Figure 1b), supercritical CO_2_ drying was carried out to produce suspended nanowires (Figure 1c).

Electrode structure and topography were inspected in a scanning electron microscope (Hitachi FE-SEM S-4800, Hitachi Ltd., Chiyoda, Tokyo, Japan) and atomic force microscope (AFM, MFP-3D, Asylum Research Inc., Santa Barbara, CA, USA) to find suitable devices. Nanowire manipulation (SmarAct 13D nanomanipulations system, SmarAct GmbH, Oldenburg, Germany) inside the SEM with etched sharp gold tips was used in order to remove other nanowires in cases where more than one nanowire was bridging the gap between the electrodes (Figure 1d,e).

The switches were electrically tested ex situ in a custom-built vacuum chamber and in SEM for visual inspection to monitor the structural stability of the switch.

Numerical calculations were carried out using FreeFem++ software (FreeFem++, version 3.5.8, http://www3.freefem.org/).

## 3. Results and Discussion

### 3.1. Fabricated CuO Nanowire-Based NEM Switch Configurations

**Figure 2:**
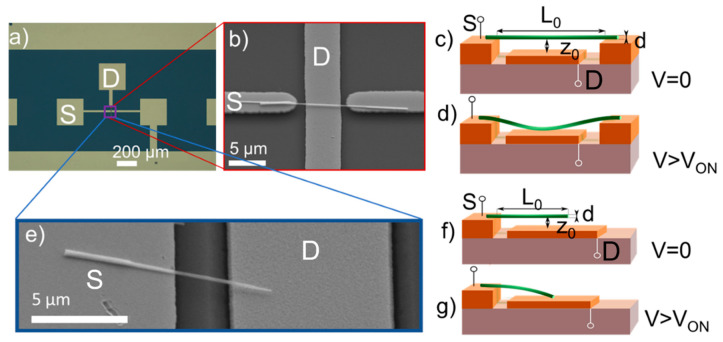
(**a**) Optical microscope image of a device connected to a micropatterned gold electrode line that joins it with other devices; (**b**) SEM image of a double-clamped single nanoelectromechanical (NEM) switch device in an array of 2 μm wide electrodes; (**c**) Schematics for a double-clamped nanowire switch in OFF and (**d**) ON state; (**e**) SEM image of a single-clamped nanowire in an electrically identical setup with wide (20 μm) DEP electrodes; (**f**) Schematics for a single-clamped nanowire switch in OFF and (**g**) ON state. Relevant design parameters shown in (**c**,**d**,**f**,**g**) are suspended nanowire length *L*_0_, nanowire diameter *d* and distance between nanowire and electrode *z*_0_.

The NEM switches that were fabricated in this work were tested in two different configurations—double-clamped and single-clamped. A single-nanowire double-clamped NEM switch device consists of a nanowire, lying flat on two gold electrodes that serve as both DEP electrodes for alignment and as the grounded switch source terminal (S) (Figure 2a–c). A single-nanowire single-clamped NEM switch has a similar configuration, but with the nanowire only fixed at one end (Figure 2a,e,f). A voltage was applied between the nanowire and lower gold drain electrode (D) to create electrostatic force *F_E_* that pulls the nanowire towards D. When the switch-ON voltage *V_ON_* was reached, a sharp current increase was detected in the circuit (Figure 2d,g). Switch-OFF voltage *V_OFF_* was registered when the sum of *F_E_* and adhesion force *F_A_* in the contact became smaller than elastic tension force *F_x_* in the nanowire and electric current fell to the current noise floor. Switch-OFF occurred at voltages that are lower than those for the switch-ON due to the presence of adhesion force *F_A_*.

### 3.2. NEM Switch Model

We modelled the forces that lead to the switch-ON and switch-OFF events in order to find the optimal operational and design parameters for repeatable NEM switching in a double-clamped setup. The balance between the electrostatic, elastic, and adhesion forces during switching were analyzed by combining the analytic and numerical approaches. The *V_ON_* was estimated for a range of nanowire diameters, corresponding to the diameter distribution acquired during synthesis. The characteristic range of nanowire diameters for CuO nanowires that were obtained by thermal oxidation in ambient air was approximately 50–200 nm [30] and the minimum distance between the side electrodes in the NEM switch was 6 μm (Figure 2b), which was chosen to ensure a reproducible photolithography process. The mechanical behavior of a double-clamped nanowire with suspended length *L*_0_ in response to an external electrostatic force can be described while using the Euler–Bernoulli, Equation (1)

(1) $$Y^{\left(4\right)}\left(\xi \right)-F_{x}\left(\xi \right)Y^{\left(2\right)}\left(\xi \right)-F_{E}\left(\xi \right)=0,\;\xi =\frac{L}{L_{0}}$$

**Figure 3:**
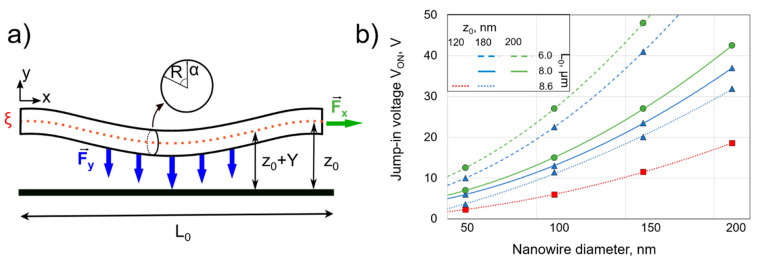
(**a**) Schematics of the relevant forces and parameters for the *V_ON_* estimation for a double-clamped nanowire with suspended length *L*_0_, diameter *d* (radius *R*) and initial distance to the electrode *z*_0_. (**b**) Calculations of *V_ON_* as a function of nanowire diameter for different distances between the nanowire and the electrode *z* and suspended lengths *L*_0_ for a Young’s modulus of 155 GPa, assuming a rigid clamping at the nanowire ends. The lines are drawn as guide to the eye. The suspended lengths are marked by line type (dashed line 6 µm, solid line 8 µm, dotted line 8.6 µm), distances are marked by colored shapes (red square corresponds to 120 nm, blue triangle to 180 nm, and green circle to 200 nm).

In (1), *Y* stands for a perpendicular displacement, *ξ*-for the normalized coordinate along the nanowire, it coincides with the x axis, when the nanowire is not deformed (voltage is not applied), *F_x_* is a tension force, and *F_E_* is the linear density of the electrostatic force (Figure 3). The derivatives over the normalized coordinate are represented as upper indices in parentheses. Equation (1) can only be used in the case of small curvatures. The range of distances between the nanowire and the electrode D was chosen in order to ensure that the switch would operate in the small curvature regime. An extra-tension force is induced at the nanowire-electrode contacts due to large curvatures, as described in [42]. It should be mentioned that all of the physical quantities with the dimensionality of length are given in units of the nanowire length, except the nanowire’s suspended length *L*_0_ itself.

The linear density of the electrostatic force is given as an integral over the corresponding circular cross section perimeter (2) (Figure 3a).

(2) $$F_{E}=\frac{4\varepsilon_{0}}{\pi EL_{0}R^{3}}\int_{0}^{2\pi }{\left(\frac{d\varphi }{dr}\right)}^{2}\cos\alpha d\alpha$$

In (2), *E* stands for the material Young’s modulus, *ε*_0_ for the permittivity of vacuum, *R* for the radius of the nanowire cross section, and *φ* for the electric potential on the surface. We consider the nanowire as an infinite conducting cylinder. The integration of electric potential over a cross-section perimeter of the nanowire is carried out in order to find the linear density of electrostatic force *F_E_*. If the displacement in axial direction is forbidden, then the nanowire will experience tension force *F_x_*. This allows for one to obtain an analytic formula for the electric potential, and, finally, using (2), for the required electrostatic force (3).

(3) $$F_{E}=\frac{16}{EL_{0}R^{4}}\frac{V^{2}\varepsilon_{0}}{\sqrt{z^{2}-R^{2}}Lnc^{2}}\;,\;c=\frac{2z^{2}}{R^{2}}\left(1-\sqrt{1-\frac{R^{2}}{z^{2}}}\right)-1$$

In (3), *z* stands for the distance from the cylinder axis to the counter electrode, but *V* for the applied voltage. To use the obtained Formula (3) in Equation (1), it is necessary to adjust the distance to the counter electrode for each cross section according to the nanowire deflection.

(4) $$z=z_{0}+Y\left(\xi \right)$$

In (4), *z*_0_ represents an initial distance from the nanowire axis to the counter electrode (Figure 3a).

The boundary conditions at the nanowire endpoints determine the tension force *F_x_* in Equation (1). If at least one of the nanowire endpoints can slide in the axial direction, then the *F_x_* is equal to zero. If they are fixed in the axial direction at both ends, then the nanowire will be under tension due to inevitable elongation when it is attracted to the counter electrode, and the tension force *F_x_* can be written as:

(5) $$F_{x}=\frac{4}{R^{2}}\left[1-\int_{0}^{1}\sqrt{1-{\left(Y^{\left(1\right)}\left(\xi \right)\right)}^{2}}\;d\xi \right]$$

In all cases, we considered the nanowire axis direction to be fixed, because of the substantial length of nanowire lying on the electrode. This leads to the following boundary conditions for the perpendicular deflection of the nanowire:

(6) $$Y\left(0\right)=Y\left(1\right)=Y^{\left(1\right)}\left(0\right)=Y^{\left(1\right)}\left(1\right)=0$$

In order to estimate the *V_ON_*, the problem that is formulated by Equations (1) and (3)–(6) has been numerically solved for a set of increasing values of electric voltage. The threshold voltage, above which the solution could no longer be found (the deformed nanowire intersects the surface of the counter electrode) is reported as *V_ON_*.

Figure 3b shows the calculated results for *V_ON_* as a function of nanowire diameter for three different suspended lengths of the nanowires (6, 8, and 8.6 µm) and three different distances between the nanowire and the electrode (120, 180, and 200 nm). The *L*_0_ and *z*_0_ values were chosen according to the fabrication method. The calculated *V_ON_* values ranged from 2 to 48 V for NEM switches while using nanowires with diameters in the range of 50–200 nm, which are typical for the used synthesis method. We use the model to find design parameters (*L*_0_, and *z*_0_) that allow for operating in the *V_ON_* range below 50 V to prevent burn-out of the nanowire and minimizing the current induced changes in the contact [8,9,22].

For the switch-OFF to occur, the elastic force of the nanowire should exceed the adhesion force in the contact. For finding the adhesion force *F_adh_*, we used the Maugis–Dugdale model adapted by Carpick et al. [43], which has been used for calculations of adhesion force in NEM switch nanowire–electrode contacts and it has shown good agreement with the experimental data [8,9,10].

The restoring elastic force *F_el_* of the nanowire in contact was found when assuming that adhesion force in contact acts as a point force applied in the middle of the beam. The equation that describes such a case is

(7) $$F_{el}=\frac{48EIz}{L_{0}^{3}}$$

where *I* is the second moment of area of the nanowire and *L*_0_–suspended length of the nanowire.

We find that, for example, for a nanowire with a diameter of 150 nm, the optimal suspended nanowire’s length is in the range of 6–8 µm and the initial distance between the nanowire and electrode *z*_0_: 120–190 nm. If these conditions are satisfied, then the calculated elastic force (5.2–8.7 × 10^−6^ N) in the nanowire exceeds the adhesion force in the contact (4.1–7.9 × 10^−6^ N), thus allowing for a successful switch-OFF and repeated operation of the NEM switch.

### 3.3. Model Comparison with Experimental NEM Switch V_ON_ Data

**Figure 4:**
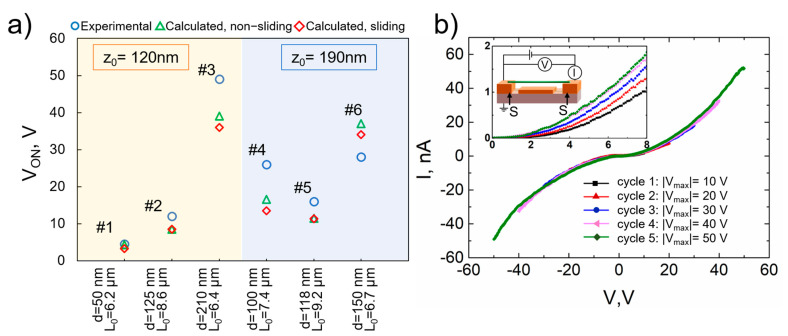
(**a**) Comparison of experimentally determined and calculated *V_ON_* values for six samples (labeled 1…6) with different nanowire diameter *d* and suspended length *L*_0_ values and two initial distances between the nanowire and the electrode *z*_0_ (120 nm and 190 nm). *V_ON_* was calculated for two different cases: with and without sliding of the nanowire ends. (**b**) I–V cycling characteristics of a single device (#6) with voltage applied between S contacts. I–V cycling starts—from maximum voltage absolute value *|V_max_|* of 10 V. The absolute value of maximum voltage is increased during every next cycle. Inset zooms in on the I–V plots for voltages up to 8 V, showing a reduction of contact resistance.

Drawing on the modelling results, we chose distances between the nanowire and electrode to be 120 and 190 nm, suspended nanowire lengths from 6.2 to 9.2 µm to fabricate, and experimentally analyze CuO NEM switches. The resolution of the photolithography limited further downscaling of suspended lengths. The experimentally determined *V_ON_* values fell within the pre-determined range for the calculated *V_ON_* values: from 4.5 V for 50 nm thin nanowire up to 49 V for 210 nm thick nanowire for different suspended lengths from 6.2 to 9.2 µm (Figure 4a). Here, all of the fabricated NEM switches are taken into account, including those that only showed one or several operation cycles due to stiction failure. The next section, will consider a typical device that showed volatile operation through many cycles.

As the ends of the nanowire are held to the electrode substrate by adhesion force only, their sliding along the surface of the substrate must be considered. The experimentally determined values for *V_ON_* were compared with calculated ones considering two different scenarios—with or without nanowire sliding at the contact (Figure 4a). For geometries that were considered in this work sliding during switch-ON can cause a decrease in *V_ON_* up to 20 % (Figure 4a). The experimentally obtained *V_ON_* values are higher in all cases. except for one (Figure 4a, device 6), which could indicate sliding in the contact in this single case. It also should be noted that the sliding could be overlooked if the Young’s moduli of the individual nanowires are higher than the average value of 155 GPa [30] used in our calculations. The sliding-induced decrease of the *V_ON_* in some cases can be observed during repeated cycling and its possible applications will be discussed later. In order to ensure a stable contact between the nanowire and the electrode, an additional fabrication step could be employed, either by lithography or electron beam induced deposition of a metal. However, this increases the complexity of the fabrication process.

### 3.4. Characterization, Testing and Optimization of Fabricated NEM Switches

In the following section, we describe contact property engineering, operation, and stability of a typical volatile NEM switch device (#6).

#### 3.4.1. Reduction of Nanowire-Electrode Contact Resistance by I–V Cycling

Nanowires that are aligned with DEP may have large contact resistances that can typically be improved by high temperature annealing or argon ion etching [44]. Here, we use an approach that was suggested by Meija et al. [22] employing I–V cycling to improve both contact electrical conductivity and its mechanical strength, which can reduce sliding in the contact. I–V cycling was carried out by applying voltage between the S-S electrodes that support the ends of the nanowire (Figure 4b inset). I–Vs were measured biasing from 0 V to 10 V and symmetrically from 0 V to −10 V with 0.1 V increment. For every next cycle, the absolute value of the maximum voltage *|V_max_ |* was increased with a 10 V step until it reached 50 V (Figure 4b). SEM analysis showed no signs of nanowire degradation, which demonstrated the suitability of CuO active elements for high voltage applications. The symmetric and nonlinear shape of the I–V curves (Figure 4b) suggests that current conduction in these nanowires is a combination of ohmic and space-charge-limited currents, as reported previously [33,45]. The current flowing through the nanowire during I–V cycling induces Joule heating at nanowire-electrode contacts. The I–V characteristics of all NEM switches examined in this work showed an increase of CuO nanowire conductivity with cycling. Figure 4b shows the results for a single NEM switch. The conductivity of the system increased almost two times during 10 cycles. This may be attributed to both enhanced contact [22] and nanowire conductivity [46].

#### 3.4.2. Initial Stages of NEM Switch Operation: *V_ON_* Stabilization

**Figure 5:**
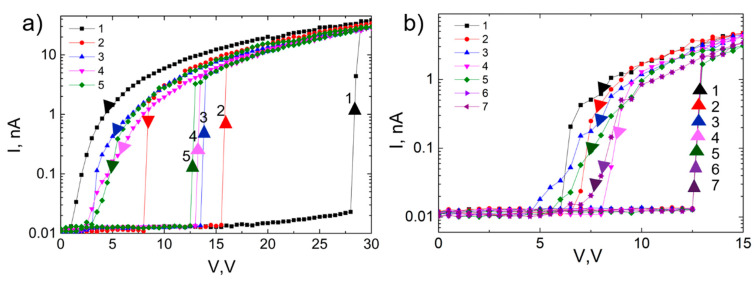
(**a**) Switch I–V cycling from 0 V to 30 V and back to 0 V shows stepwise decrease of *V_ON_* for every next cycle (cycles from 1 to 5) until (**b**) reaching a stable NEM switch operation regime with constant *V_ON_* at 12.5 V and *V_OFF_* at 4.5–8 V (cycles 1 to 7: I–V cycling from 0 V to 15 V and back to 0 V). Upwards arrows mark transition to ON state, downwards arrows–transition to OFF state. For this device nanowire’s diameter *d* = 150 nm, *L*_0_ = 6.7 µm, and *z*_0_ = 190 nm.

The NEM switch operation was tested by applying the same voltage biasing scheme as during the reduction of contact resistance between the nanowire S and the lower electrode D (Figure 2). This approach is valuable for analyzing the stability of *V_ON_* and *V_OFF_* values. Figure 5a shows the first five cycles of the NEM switch, when it is cycled between 0–30 V. In the first I–V (1), the switch-ON occurs at 28 V and switch-OFF at 1 V. In the second cycle, the *V_ON_* decreases from 28 V to 15.5 V. During the next three cycles, it gradually decreases towards 12.5 V. As a result of the cycling, the value of *V_OFF_* slightly increases from 1 V to 2.5–3.5 V. The initial decrease of *V_ON_* may be attributed to the nanowire-upper electrode contact modification, which can result in the release of internal mechanical stresses, as well as sliding along the surface. This prevents the nanowire’s return to its original position, and it remains in a slightly deformed shape. In every next voltage cycle, the nanowire slides again until a new stable equilibrium position is found for stable NEM switch operation. A similar step-wise decrease of initial *V_ON_* value has been reported for the CNT bundle-based NEM switch, where the authors suggest a slipping of the CNT bundle on the support as the possible explanation [47]. This can be contrasted to the gradual voltage decrease observed in [27], which was interpreted as a removal of an outer layer of the nanowire material. The relatively constant ON current level suggests that mechanical effects are responsible for the observed changes in the NEM switch characteristics and there is not much dissipation of electrical power in the nanowire-electrode contact. After five cycles the *V_ON_* value stabilized and a stable ON-OFF operation of NEM switch followed (Figure 5b), where the switch-ON occurred at 12.5 V and switch-OFF at 4.5–8 V. A decrease of the hysteresis loop may be beneficial for NEM switch operation, as it decreases energy dissipation. The switch-ON is much more abrupt than the switch-OFF, which can be explained by the relatively large contact area of the double-clamped nanowire that needs to detach from the electrode as the voltage is decreased. The differences in the *V_OFF_* values between cycles can be explained by variations in contact area. Additionally, the sliding of the nanowire along the contacts can be deliberately employed in order to fine-tune the NEM switch *V_ON_* to a desired value. When it is reached, the active element can be mechanically fixed while using an additional fabrication step (e.g., by lithography).

**Figure 6:**
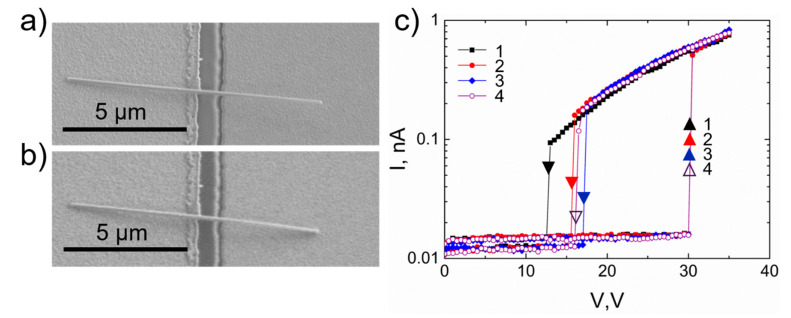
CuO nanowire-based NEM switch in a single-clamped configuration. SEM image of the NEM switch in (**a**) OFF state, (**b**) ON state; (**c**) Switch I–V cycling showing sharp transition to ON (at 30 V) and OFF (at 13–17 V) states, four switching cycles are shown. Upwards arrows mark transition to ON state, downwards arrows–transition to OFF state. Dimensions for this device: suspended length *L*_0_ = 3 μm, diameter *d* = 80 nm, and the initial distance between nanowire and electrode *z*_0_ = 190 nm.

Figure 6a,b shows an example of a single-clamped NEM switch. The geometry of this switch was chosen according to the previous theoretical and experimental work conducted by our group [8,9,12]. *V_ON_* for this device was 30 V and *V_OFF_* 13–17 V. In contrast to the double-clamped case, the transition to ON/OFF states is sharp both for ON and OFF switching, which can be explained by the smaller contact area and faster detachment from the contact electrode. The calculated adhesion force [43] for this single-clamped nanowire (2 × 10^–7^ N) is an order of magnitude smaller than for a double-clamped case, which allows for a sharper transition between the ON and OFF states. The smaller adhesion force can be explained by two orders of magnitude smaller effective contact area (78 nm^2^ for a single-clamped and 7850 nm^2^ for a double-clamped case).

#### 3.4.3. Cycling of NEM Switch

**Figure 7:**
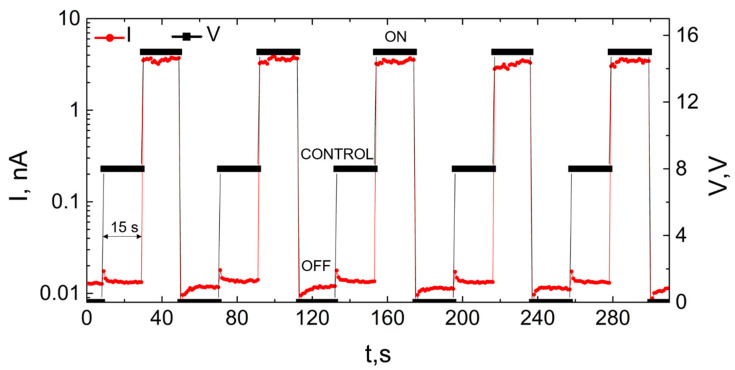
Variation of NEM switch current levels (red circles) at different applied voltages (black squares) of 0 V (OFF), 8 V (OFF, control), 15 V (ON) during repeatable switching. For 0 V and 8 V, the current stays at the noise level, whereas ON current reaches nA level, when applied voltage is 15 V.

Continued cycling of a double-clamped NEM switch was performed in order to demonstrate the stability of the NEM switch operational parameters. Voltage was applied in square pulses, alternating between 15 V (ON state), 0 V (OFF state), and 8 V (detachment test). The latter voltage value was chosen to be smaller than the switch-ON voltage, but larger than the non-conductive gap region observed in I–V characteristics of the nanowire (Figure 4b) in order to detect the nanowire detachment without visual inspection. The expected values for the current at each state were the following: signal in nA range for 15 V applied voltage and system noise for both 0 V and 8 V. Figure 7 shows a fragment of a typical ON/OFF I(t) cycling, where the switch remains for 15 s in each of the states.

## 4. Conclusions

In summary, we demonstrate the first fabrication and volatile operation of a NEM switch on-chip that employs bottom-up synthesized semiconductor nanowires as the active elements. It is shown that bottom-up synthesized CuO nanowires can serve as a robust and reliable material for the active element fabrication. By combining conventional lithography, dielectrophoresis, and nanomanipulations, as shown in this work, the fabrication yield can be increased, and device operation assessed more quickly.

We propose a theoretical model for estimating switch-ON voltage, adhesion, elastic force, and nanowires sliding dependence on nanowire’s suspended length, diameter, and distance to the electrode in order to obtain parameters for successful volatile device operation. The model was validated by fabricating NEM switches with the predetermined geometry and testing their operation in gateless double-clamped setup. The calculated *V_ON_* values were close to those obtained experimentally, and volatile operation was achieved. DEP alignment was successfully employed to also fabricate single-clamped NEM switches.

Both single- and double-clamped CuO nanowire-based switches exhibit abrupt switch-ON, requiring approximately 0.5 V to transit from OFF to ON state. Single-clamped devices show increased switching speeds from the ON to OFF state, which suggests that they can offer faster operation and better energy efficiency than double-clamped configuration switches. However, the demonstrated bottom-up fabrication approach using DEP is currently more suitable for scalable fabrication of double-clamped switches, due to the efficient control of suspended lengths of the nanowires by DEP parameters and electrode geometry. The scalable fabrication of single-clamped switches will require improved control over the suspended length of the nanowire. We also suggest that voltage cycling can be used as a step in NEM switch fabrication to stabilize *V_ON_*. Further research will be devoted to improving the nanowire-electrode contact in order to improve NEM switch operational characteristics.

## Data Availability

The data presented in this study are available on request from the corresponding author.
